# Improving difficult direct laryngoscopy prediction using deep learning and minimal image analysis: a single-center prospective study

**DOI:** 10.1038/s41598-024-65060-x

**Published:** 2024-06-20

**Authors:** Jong-Ho Kim, Hee-Sun Jung, So-Eun Lee, Jong-Uk Hou, Young-Suk Kwon

**Affiliations:** 1 Division of Big Data and Artificial Intelligence, Institute of New Frontier Research, Chuncheon Sacred Heart Hospital, Hallym University College of Medicine, Chuncheon, 24253 Republic of Korea; 2 Department of Anesthesiology and Pain Medicine, Chuncheon, Sacred Heart Hospital, 77 Sakju-ro, Chuncheon-si, Gangwon-do 24253 Republic of Korea; 3 Division of Software, Hallym University, 1, Hallymdaehak-gil, Chuncheon-si, Gangwon-do 24252 Republic of Korea; 4 Department of Intelligence Computing, Hanyang University, Seoul, Republic of Korea

**Keywords:** Medical research, Risk factors

## Abstract

Accurate prediction of difficult direct laryngoscopy (DDL) is essential to ensure optimal airway management and patient safety. The present study proposed an AI model that would accurately predict DDL using a small number of bedside pictures of the patient’s face and neck taken simply with a smartphone. In this prospective single-center study, adult patients scheduled for endotracheal intubation under general anesthesia were included. Patient pictures were obtained in frontal, lateral, frontal-neck extension, and open mouth views. DDL prediction was performed using a deep learning model based on the EfficientNet-B5 architecture, incorporating picture view information through multitask learning. We collected 18,163 pictures from 3053 patients. After under-sampling to achieve a 1:1 image ratio of DDL to non-DDL, the model was trained and validated with a dataset of 6616 pictures from 1283 patients. The deep learning model achieved a receiver operating characteristic area under the curve of 0.81–0.88 and an F1-score of 0.72–0.81 for DDL prediction. Including picture view information improved the model’s performance. Gradient-weighted class activation mapping revealed that neck and chin characteristics in frontal and lateral views are important factors in DDL prediction. The deep learning model we developed effectively predicts DDL and requires only a small set of patient pictures taken with a smartphone. The method is practical and easy to implement.

## Introduction

Airway management is a cornerstone of emergency care, anesthesia, and critical care, essential for safeguarding patient safety during procedures that impair natural ventilation, such as those necessitating intubation^1,2^. Effective management is paramount not only in direct airway-related interventions but also in preparing for potential complications in various medical conditions where respiratory status may be at risk for a difficult airway or difficult airway management^3^. Predicting difficult airways is crucial for preemptive planning and adopting tailored strategies to mitigate risks like airway obstruction or failure. Early identification allows for mobilizing specialized equipment and skilled personnel, enhancing successful intubation and minimizing risks such as failed intubation, esophageal intubation, and hypoxia^4^.

Direct laryngoscopy is a crucial medical procedure that involves using a laryngoscope to directly visualize the vocal cords and the entrance to the trachea. This technique is essential for performing tracheal intubation, commonly required in anesthesia and emergency settings to secure the airway. Difficult Direct Laryngoscopy (DDL) occurs when the best achievable view of the glottis is less than optimal, characterized by a Cormack-Lehane grade of 3 or 4, for example, indicating poor visualization of the glottis. Such suboptimal views can significantly compromise patient safety if not anticipated and managed appropriately. Accurately predicting DDL is vital for selecting the appropriate intubation technique and enhancing patient safety. However, this prediction is complicated by the complex nature of airway anatomy and the interaction of various patient-specific factors. Critical anatomical predictors of DDL include limited thyro-mental distance (ideally exceeding 5 cm), abnormal jaw positioning such as retrognathia or micrognathia, restricted neck extension, and insufficient mouth opening (minimum of three finger breadths). These features are reported in various literature as essential indicators for anticipating difficult airway management scenarios.

However, accurately predicting difficult direct laryngoscopy (DDL) poses significant challenges. Contrary to the expectations, commonly used bedside assessment tests are well-documented to have low sensitivity and are generally poor predictors of airway difficulty. This established viewpoint, as reported in the Cochrane Database of Systematic Reviews 2018^5^, emphasizes that these tests often fail to identify all potential cases of a difficult airway reliably. The misconception of their high sensitivity continues despite substantial evidence, including historical research like that of Wilson ME^6^, which has demonstrated their limitations in predicting DDL for over two decades. This discrepancy between theoretical effectiveness and actual clinical utility highlights a significant gap, necessitating further research to develop more reliable and sensitive tools for predicting difficult airways.

AI-based approaches for DDL prediction have shown promise, utilizing machine learning techniques like computer vision and image analysis to accurately predict DDL^7–9^. The integration of AI in airway management can enhance decision-making, optimize patient outcomes, and improve efficiency^10–14^. However, previous AI-based approaches for predicting difficult direct laryngoscopy (DDL) often faced significant constraints, being tailored to specific surgical environments or requiring extensive sets of image data and the measurement of airway-related features. These constraints limited the practical application and scalability of AI solutions across diverse clinical settings. In contrast, our model utilizes a minimal set of readily available bedside images, making it broadly applicable to all patients. This design significantly enhances the model's practicality and adaptability, addressing the limitations observed in previous studies.

## Methods

This single-center and prospective study was conducted at Sacred Heart Hospital in Chuncheon, South Korea, from September 11, 2019, to August 31, 2021. The study was approved by the Institutional Review Board of Chuncheon Sacred Heart Hospital (IRB No. 2019-08-015). All study procedures were performed in accordance with relevant guidelines and regulations, adhering to the principles stipulated in the Declaration of Helsinki. The clinical trial protocol was registered at https://cris.nih.go.kr. Informed consent was obtained from all participants, including patients and anesthesiologists involved in the study. Verbal informed consent for the publication of identifying information/images in an online open-access publication was obtained from subjects and/or their legal guardians.

### Inclusion and exclusion criteria

**Figure 1:**
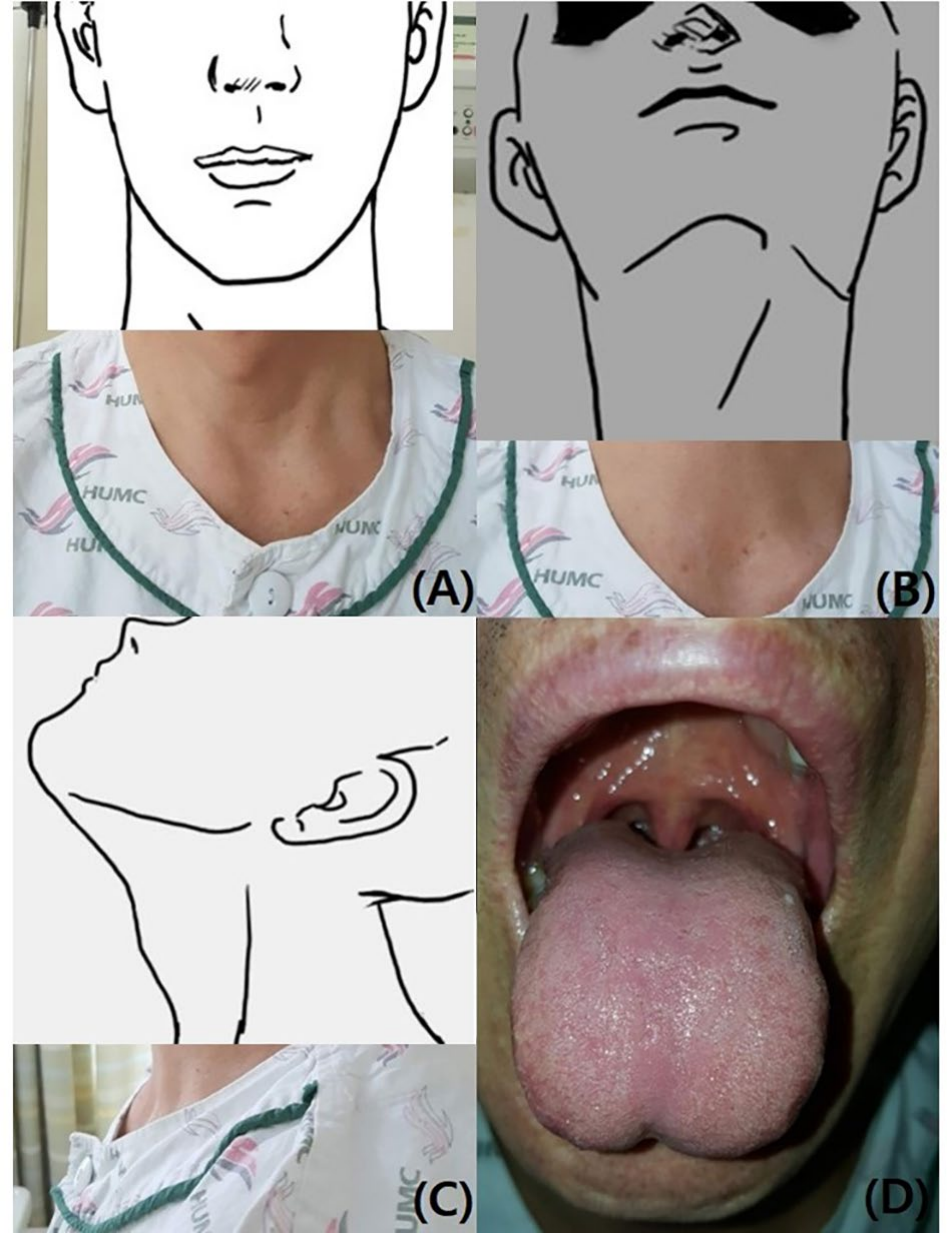
Four types of pictures. (**A**) Frontal view, (**B**) Frontal-neck extension view, (**C**) Lateral view, (**D**) Mouth opening view.

Adult patients (age > 18 years) scheduled for endotracheal intubation under general anesthesia were eligible for study inclusion. We excluded patients who were intubated outside the operating room, unconscious, had major external facial or neck abnormalities, laryngeal abnormalities, or tumors. Only patients who adhered to NPO (nil per os) guidelines, ensuring an empty stomach, were included, thus excluding those with full stomachs. However, patients undergoing cervical spine surgery and some emergency surgeries were included in the study. We collected photographs in four views: frontal, lateral, frontal-neck extension, and open mouth. The examples are shown as drawn images in Fig. 1. The identification of major external facial or neck abnormalities was entrusted to the experienced anesthesiology staff involved in the research. These practitioners used their clinical expertise to discern abnormalities that could influence airway management, ensuring a consistent and informed approach to participant inclusion.

### Laryngoscopy

Laryngoscopy is a medical procedure that plays a pivotal role in airway management, particularly preceding intubation processes. This procedure involves the use of a laryngoscope to expose and visualize the larynx, providing a clear, line-of-sight view of the vocal cords and the entrance to the trachea. The primary objective of laryngoscopy is to facilitate a safe and efficient intubation by allowing healthcare professionals to guide the endotracheal tube into the trachea with minimal risk of injury to the patient.

Standard Macintosh metallic single-use disposable laryngoscope blades (INT; Intubrite LLC, Vista, CA, USA) were employed. To ensure sufficient muscle relaxation, all patients received a dose of rocuronium, administered at 0.6–0.8 mg/kg, followed by a 3-min waiting period before proceeding with tracheal intubation in the supine position^15^. To ensure a standardized depth of anesthesia suitable for intubation, propofol was administered, with additional opioids provided as necessary based on the patient's condition. Anesthesia depth was monitored using appropriate devices to confirm adequate general anesthesia before proceeding with intubation. The Cormack–Lehane grading system was used to assess laryngeal views before any external laryngeal manipulation was performed. However, external laryngeal manipulation was applied^16^ if necessary after the initial grading to improve the laryngeal view and aid in the intubation process.

This method provided a consistent approach to achieving the requisite level of muscle relaxation for safe intubation. Patient positioning was standardized by placing all patients in the supine position, utilizing a standard-sized pillow to support uniform head and neck alignment. This procedure aimed to minimize variability in patient positioning during intubation, contributing to the overall standardization of the intubation process despite the challenges presented by the lack of universal neuromuscular monitoring capabilities.

The direct laryngoscopy views were classified as follows in accordance with the Cormack–Lehane grading system:

- Grade 1: most of the glottic opening is visible.
- Grade 2: only the posterior portion of the glottis or only the arytenoid cartilages are visible.
- Grade 3: only the epiglottis but no portion of the glottis is visible.
- Grade 4: neither the glottis nor the epiglottis is visible.

To ensure clarity and precision in our study, DL was defined based on the Cormack-Lehane grading system, where a grade of 3 or 4 indicates a situation where the glottis is not fully visible or not visible at all, respectively. The Cormack–Lehane grading system was used to assess laryngeal views before any external laryngeal manipulation was performed. However, to facilitate successful intubation, external laryngeal manipulation was applied if necessary after the initial grading. This grading system is widely accepted and utilized in numerous studies as a reliable indicator for assessing laryngoscopy difficulty^17–19^. Consequently, patients exhibiting a Cormack-Lehane grade of 1 or 2 were categorized as NDDL, indicating a relatively easier laryngoscopic view and procedure. If the vocal cord was not visualized during the first attempt, a practitioner manipulated the laryngoscope to obtain the best view of vocal cord exposure. In cases of failed intubation, we prioritized patient safety by avoiding repeated attempts using the same method and instead considered alternative and appropriate approaches.

To reduce variability in the Cormack-Lehane grading among anesthesiologists, we conducted a training session for all participating anesthesiologists before the commencement of our study. The session focused on reviewing a wide range of laryngeal view cases, emphasizing the importance of a unified understanding and application of grading criteria. This standardization aimed to ensure consistency in evaluating laryngeal views across practitioners. Although the training was thorough, we did not formally evaluate the consistency of Cormack-Lehane grading between anesthesiologists post-training.

The final Cormack-Lehane grade was recorded by seven attending anesthesiologists and six resident anesthesiologists. The attending anesthesiologists had significant experience in airway management, with the least experienced having at least seven years of experience at the start of the study. The other attending anesthesiologists had 11, 13, 14, 18, 24, and 28 years of experience, respectively. Although the training was thorough, we did not formally evaluate the consistency of Cormack-Lehane grading between anesthesiologists post-training; here is a brief overview of each practitioner type included in our study:

Attending Anesthesiologists: Board-certified physicians who have completed their residency training in anesthesiology and possess comprehensive knowledge and experience in airway management, including tracheal intubation.

Anesthesiology Residents: Physicians in training who are in the process of completing their residency in anesthesiology. Their experience with tracheal intubation varies between 1 to 5 years.

Moreover, practitioner experience levels were categorized as 1–2 years, 2–3 years, 3–4 years, 4–5 years, and over 5 years, reflecting the variability in difficulty determination contingent on the experience of the practitioners conducting tracheal intubation. This categorization was utilized as supplementary information to enhance the model's predictions.

### Photography protocol and quality control

One of the researchers visited the patients and took pictures either the day before or on the day of the surgery. All pictures were at the patients’ bedside while they were in a sitting position as much as possible. If the patient was lying down or in a semi-fowler’s position, the pictures were taken without changing their position. All images were taken with a smartphone (Samsung Galaxy S6 Edge; Samsung, Suwon, Republic of Korea).

In our study, instead of capturing exactly four photographs per patient, we aimed to collect four types of photographs for each participant: frontal, frontal with neck extension, lateral, and open mouth views. These images were utilized to develop our deep learning model. It's important to note that the collection process did not limit us to only four images per patient. In instances where the initial photos from either the frontal or lateral perspectives slightly deviated from the desired angle, additional photographs were taken to secure as accurate a representation as possible. Consequently, this approach led to some patients contributing more than the standard set of four photographs to our dataset. This methodology was adopted to enhance the robustness and accuracy of our deep learning model by providing a more varied and extensive dataset.

In designing our study, we acknowledged the variability present in clinical settings that could affect the conditions under which photographs are taken, such as the distance from the patient, lighting conditions, and the background environment. Given the diverse nature of clinical environments and the variable conditions under which healthcare professionals operate, we deliberately chose not to standardize these aspects of photography. This decision was aimed at capturing a wide array of images that reflect real-world scenarios, thereby enhancing the practical applicability of our deep learning model across diverse clinical settings.

Our model was trained to accurately predict Difficult Direct Laryngoscopy (DDL) using images captured under various conditions, including different patient positions, levels of lighting brightness, and backgrounds. Regarding quality control, our methodology did not involve the exclusion of photographs based on technical problems or quality issues, except in cases where the image clarity was insufficient to discern relevant anatomical features.

### Data preprocessing and deep learning

*Baseline deep learning encoder:* We utilized the EfficientNet-B5 model, widely acknowledged for its efficacy in image classification tasks, as our base network^20^. This pre-trained model, derived from the ImageNet database, is particularly noted for its smaller parameters and reduced computational cost compared to existing ImageNet models. The EfficientNet-B5 model comprises 30 M parameters and is capable of executing 9.9B floating-point operations per second. To tailor the network outputs for our specific application, we modified the last classifier module of the EfficientNet-B5 to include a new classifier. This classifier features a fully connected layer with two output nodes, supplemented by dropout regularization.

**Figure 2:**
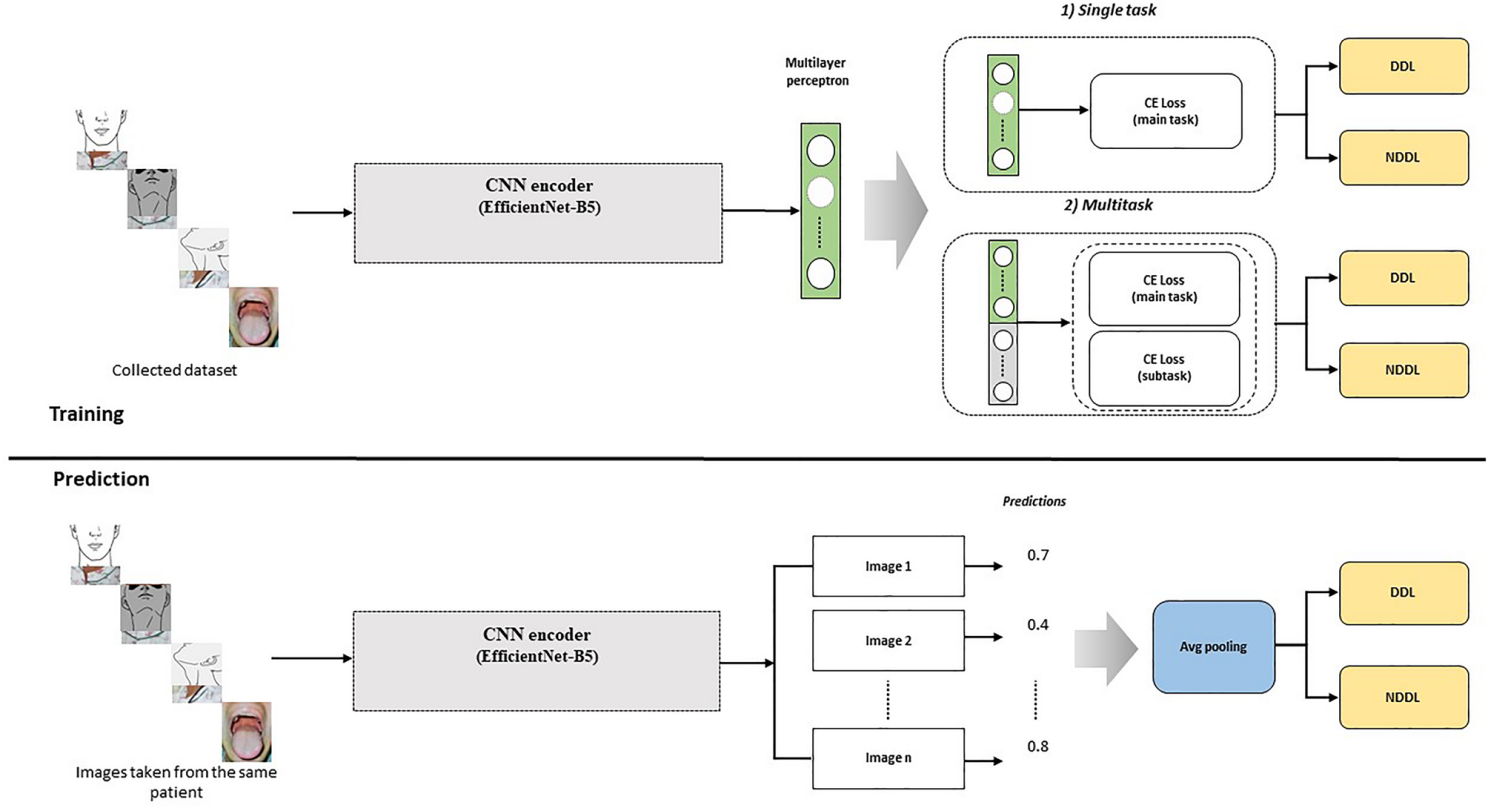
Overall architecture of the proposed deep learning framework. Avg: average, CE Loss: categorical cross-entropy loss, CNN: convolutional neural network, DDL: direct difficult laryngoscopy, NDDL: non-difficult laryngoscopy. EfficientNet uses Mobile Inverted Bottleneck (MBConv) layers (Sandler, Mark, et al. "Mobilenetv2: Inverted residuals and linear bottlenecks." Proceedings of the IEEE conference on computer vision and pattern recognition. 2018.), which are a combination of depth-wise separable convolutions and inverted residual blocks. Additionally, the model architecture uses the Squeeze-and-Excitation (SE) optimization to further enhance the model's performance.

*Multitask formulation:* Embracing multitask learning facilitates inductive transfer between tasks, leveraging additional information—such as picture view information^21^—to enhance model performance. We adopted a hard parameter sharing technique to mitigate overfitting risks. The multitask learning objective is formulated as follows:

1 $$ {\mathcal{L}} = \beta \cdot {\mathcal{L}}_{CE} (C_{\theta } \left( {E\left( I \right)} \right) + \left( {1 - \beta } \right) \cdot {\mathcal{L}}_{CE} \left( {C_{{\theta^{\prime}}} \left( {E_{C} \left( I \right)} \right)} \right), $$

where ${\mathcal{L}}_{CE}$, ${C}_{\theta }$, and ${C}_{{\theta }{\prime}}$ denote categorical cross-entropy loss, main task classifier, and subtask classifier for multitask learning, respectively. $E\left(I\right)$ and $I$ denote the image encoder (EfficientNet-B5) and an input image, respectively. Equation 1 represents multitask learning, which enables simultaneous improvement in the performance of the image encoder by training a subtask alongside the main task. In this study, the subtask involves predicting the 'career information of tracheal intubation practitioners,' serving as auxiliary information that can indicate trends between input images and their predicted values. The weight between tasks, beta, is set experimentally at 0.9, indicating that the loss function for the main task is weighted nine times more heavily. This ratio is determined to provide an appropriate synergy to our main task. Additionally, the categorical cross-entropy loss, a widely used baseline loss function for solving image classification problems, allows our backbone model, EfficientNet-B5, to be optimally trained for classification tasks. The overall structure is depicted in Fig. 2. We use the weight value $\beta$ = 0.9 to focus on the main task.

*Training and validation strategy*: Training was executed on an Nvidia Geforce RTX 3090 graphic processor (Nvidia, Santa Clara, CA, USA). The batch size and training epoch were established at 32 and 20, respectively. We applied a learning rate of 3e-5, accompanied by a cosine annealing warm restart scheduler and Adam optimization^22^. Data augmentation techniques were employed to simulate training with an expanded dataset, aiming to avert model overfitting and foster generalization performance. Utilizing the Albumentations library—a swift and versatile data enrichment framework^23^—we applied image resizing and contrast-limited adaptive histogram equalization^24^, a method designed to adjust image contrast by expanding the histogram at both ends when the image pixels congregate around a specific value range. Adjustments to image color were also feasible; however, the color alterations in patients with intubation issues were disregarded as deemed irrelevant. Additionally, image flip and rotation were excluded to prevent model training distractions, as such transformations could warp the patient’s positional information.

We amassed 18,163 pictures from 3053 patients. The compilation included 3310 DDL and 14,853 NDDL images. The distribution of images among the frontal, lateral, frontal-neck extension, and open-mouth views were 4838, 4496, 5311, and 3518, respectively. Utilizing under-sampling, we matched NDDL and DDL images in a 1:1 ratio. Post-matching, the training dataset consisted of 6616 pictures (3308 NDDL and 3308 DDL images) across 1283 patients (771 NDDL and 512 DDL patients), with fourfold cross-validation implemented during training. The distribution of NDDL and DDL images included in each fold was as follows: Fold 0: NDDL, 831; DDL, 828. Fold 1: NDDL, 840; DDL, 838. Fold 2: NDDL, 814; DDL, 824. Fold 3: NDDL, 823; DDL, 818. To ensure consistency, images associated with the same patient number were assigned to the same fold, thus preventing overlap during training and validation phases. Each patient’s images were evaluated individually using the neural network to generate predictions. The predictions from the various views of the same patient were then combined using average pooling to derive a final prediction for that patient. As illustrated in Fig. 2, each patient’s images were evaluated individually to derive a prediction. Subsequently, these results were amalgamated through average pooling to formulate a final decision. We determined the optimal threshold for each fold and computed the score accordingly. The evaluation performance was demonstrated through precision, recall (sensitivity), F1-score, and receiver operating characteristic area under the curve (ROC AUC) metrics, with 'support' referring to the class-specific data volume in our dataset. 'Macro avg' was calculated using the arithmetic mean of the scores without consideration for class ratio, and the 'weighted average value' was determined by averaging all per-class scores with class ratio consideration. All data preprocessing, model training, and validation processes were conducted using Python (www.python.org, version 3.8.13) and PyTorch (version1.5.0, Facebook's AI Research lab, Menlo Park, CA, US) library.

The original model predicts DDL using only the patient's photographs. However, additional considerations included providing information about each photograph's specific view type, such as whether the photo is a frontal view, open mouth view, lateral view, or frontal-neck extension view. This supplementary information helps improve the model's ability to discern tracheal intubation difficulties.

### Ethics approval and consent to participate

This study protocol was approved by the institutional review board of Chuncheon Sacred Hospital (CHUNCHEON 2018-12-006). Informed consent was obtained from all participants in the study.

## Results

### Baseline experiment

This study initially included 3,305 participants, but 252 were excluded due to errors or incomplete records These images were captured using, resulting in a total of 3,053 participants being included in the analysis. Table 1 presents the baseline characteristics of patients included in this study prior to undersampling. The distribution of images and patients by classification and fold is summarized in Table 2.

**Table 1:** The characteristics of patients.

	N = 3053
Age, year (Interquartile range)	58 (45–68)
Male, number (%)	1681 (55.1)
Height, cm (Interquartile range)	163 (155.9, 170)
Weight, kg (Interquartile range)	65.8 (57.3–74.7)
Body mass index (Interquartile range)	24.8 (22.6–27.3)
Emergency surgery, number (%)	107 (3.5)

**Table 2:** Distribution of images and patients by classification and Fold.

Fold	Total Images (n = 6616)	NDDL Images (n = 3308)	DDL Images (n = 3308)	NDDL Patients	DDL Patients	Total Patients
0	1659	831	828	771	512	1283
1	1678	840	838			
2	1638	814	824			
3	1641	823	818			

DDL, difficult laryngoscopy; NDDL, non-difficult laryngoscopy.

First, we determined DDL and NDDL cases using 6616 pictures from 1288 patients after undersampling. After training, the prediction probability was averaged to obtain the results for each patient. Table 3 presents the results of the cross-validation performance of the deep learning model using only image data for predicting DDL in the fourth fold. The table encompasses several key metrics to assess the effectiveness and accuracy of the model, including Precision, Recall, F1-score, and ROC AUC. The table also includes the Confusion Matrix, which is detailed with counts of True Positives, False Negatives, False Positives, and True Negatives. These measures collectively provide a comprehensive view of the model’s predictive performance and its reliability in differentiating between NDDL and DDL scenarios based on image data alone.

**Table 3:** Cross-validation performance metrics of the deep learning model using image data for predicting difficult laryngoscopy.

		Precision	Recall	F1-score	Support	ROC AUC	Confusion Matrix for Predicting DDL	
Fold 0	NDDL	0.82	0.78	0.8	831	0.86	TP	671
	DDL	0.79	0.82	0.81	828		FN	157
	Macro avg	0.8	0.8	0.8	1659		FP	168
	Weighted avg	0.8	0.8	0.8	1659		TN	663
Fold 1	NDDL	0.8	0.78	0.79	840	0.86	TP	529
	DDL	0.78	0.8	0.79	838		FN	311
	Macro avg	0.79	0.79	0.79	1678		FP	86
	Weighted avg	0.79	0.79	0.79	1678		TN	752
Fold 2	NDDL	0.7	0.88	0.78	814	0.82	TP	651
	DDL	0.84	0.63	0.72	824		FN	173
	Macro avg	0.75	0.75	0.75	1638		FP	194
	Weighted avg	0.75	0.75	0.75	1638		TN	620
Fold 3	NDDL	0.78	0.9	0.84	823	0.87	TP	638
	DDL	0.88	0.75	0.81	818		FN	180
	Macro avg	0.82	0.82	0.82	1641		FP	140
	Weighted avg	0.82	0.82	0.82	1641		TN	683

avg, average; DDL, difficult laryngoscopy; FN, false negative; FP, false positive; NDDL, non-difficult laryngoscopy; ROC AUC, Receiver operating characteristic area under curve; TN, true negative; TP, true positive.

### Experiment results based on multitask formulation

In this study, we employed multitask learning to enhance model performance by incorporating subtasks that utilized both view type and practitioner type information. The results of this approach are detailed in Table 4, which outlines the performance of the deep learning model using image data augmented with these additional features. Practitioners were categorized based on their years of clinical experience into five groups: 1–2 years, 2–3 years, 3–4 years, 4–5 years, and over 5 years. The number of patients assigned to each category was 226, 253, 311, 36, and 457, respectively. Table 5 summarizes the distribution of direct difficult laryngoscopy cases encountered by anesthesiologists, categorized by their level of experience.

**Table 4:** Cross-validation performance of the multitasking deep learning model using picture view and practitioner experience information for predicting difficult direct laryngoscopy.

Additional Information			Precision	Recall	F1-score	Support	ROC AUC	Confusion Matrix for Predicting DDL	
Picture view	Fold 0	NDDL	0.78	0.87	0.83	831	0.87	TP	629
		DDL	0.86	0.76	0.8	828		FN	102
		Macro avg	0.82	0.82	0.81	1659		FP	199
		Weighted avg	0.82	0.82	0.81	1659		TN	729
	Fold 1	NDDL	0.77	0.8	0.79	840	0.86	TP	647
		DDL	0.79	0.77	0.78	838		FN	172
		Macro avg	0.78	0.78	0.78	1678		FP	193
		Weighted avg	0.78	0.78	0.78	1678		TN	666
	Fold 2	NDDL	0.75	0.9	0.82	814	0.87	TP	585
		DDL	0.87	0.71	0.78	824		FN	87
		Macro avg	0.81	0.8	0.8	1638		FP	239
		Weighted avg	0.81	0.8	0.8	1638		TN	727
	Fold 3	NDDL	0.74	0.94	0.83	823	0.88	TP	548
		DDL	0.92	0.67	0.77	818		FN	48
		Macro avg	0.83	0.8	0.8	1641		FP	270
		Weighted avg	0.83	0.8	0.8	1641		TN	775
Practitioner Experience Information	Fold 0	NDDL	0.81	0.8	0.8	831	0.86	TP	671
		DDL	0.8	0.81	0.81	828		FN	157
		Macro avg	0.8	0.8	0.8	1659		FP	168
		Weighted avg	0.8	0.8	0.8	1659		TN	663
	Fold 1	NDDL	0.71	0.9	0.79	840	0.81	TP	529
		DDL	0.86	0.63	0.73	838		FN	311
		Macro avg	0.78	0.76	0.76	1678		FP	86
		Weighted avg	0.78	0.76	0.76	1678		TN	752
	Fold 2	NDDL	0.78	0.77	0.77	814	0.85	TP	651
		DDL	0.77	0.79	0.78	824		FN	173
		Macro avg	0.78	0.78	0.78	1638		FP	194
		Weighted avg	0.78	0.78	0.78	1638		TN	620
	Fold 3	NDDL	0.8	0.71	0.75	823	0.84	TP	638
		DDL	0.74	0.82	0.78	818		FN	180
		Macro avg	0.77	0.77	0.76	1641		FP	140
		Weighted avg	0.77	0.77	0.76	1641		TN	683

avg, average; DDL, difficult laryngoscopy; FN, false negative; FP, false positive; NDDL, non-difficult laryngoscopy; ROC AUC, Receiver operating characteristic area under curve; TN, true negative; TP, true positive.

**Table 5:** Number of practitioners and their experiences with direct difficult laryngoscopy based on the experience level of anesthesiologists.

Career in airway management	Number of practitioners (n = 13)	Number of Patients (n = 1283)	Number of DDL (%)
1–2 years	2	226	81 (35.8)
2–3 years	2	263	116 (45.8)
3–4 years	1	311	147 (47.3)
4–5 years	1	36	10 (27.8)
Over 5 years	5–10 years: 1 10–20 years: 4 Over 20 years: 2	457	158 (34.6)

DDL, difficult laryngoscopy.

### Gradient-weighted class activation mapping

**Figure 3:**
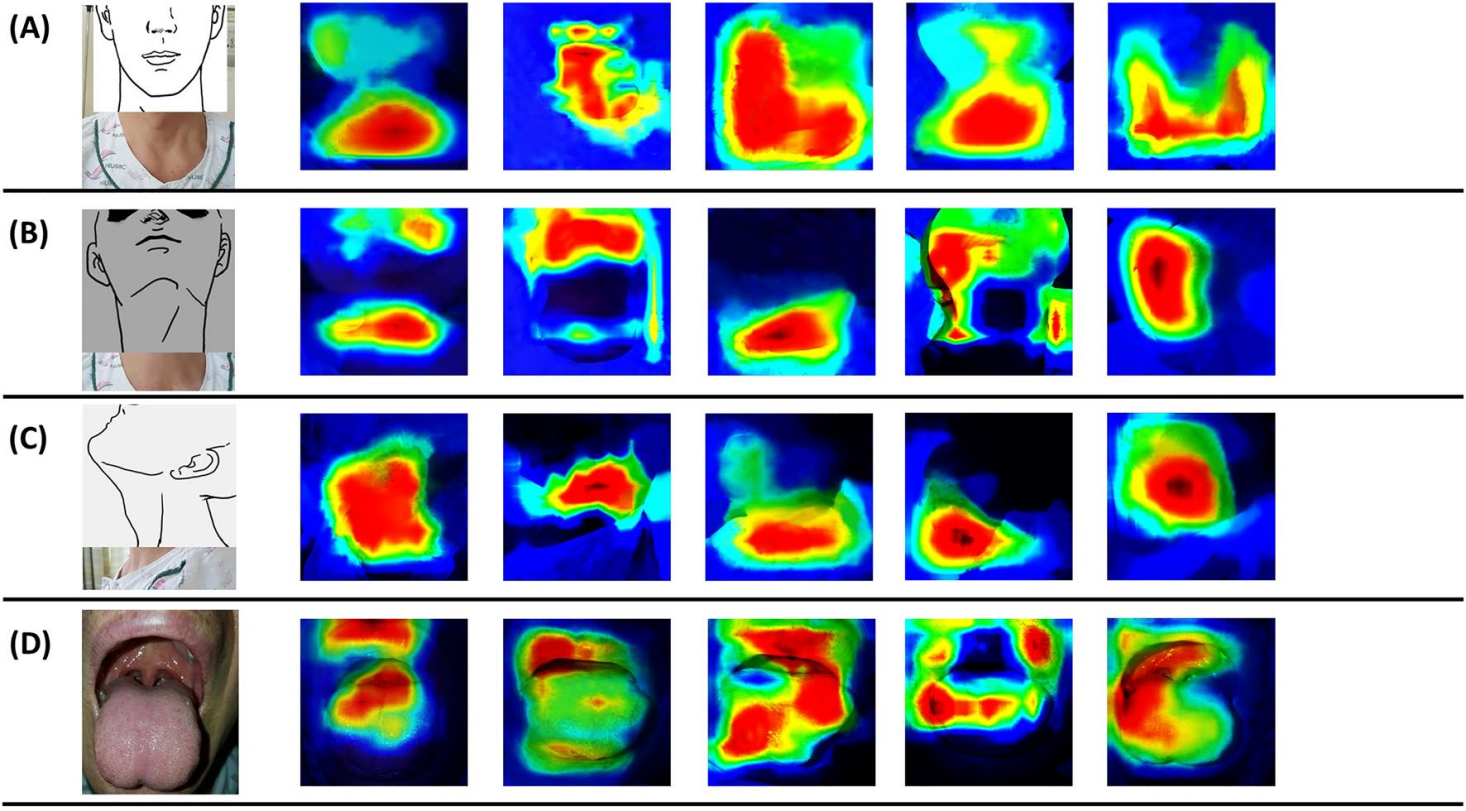
Gradient-weighted Class Activation Mapping (Grad-CAM) visualizations for patients with a high probability of DDL. Each row represents a different laryngoscopic view: (**A**) Frontal view, (**B**) Frontal-neck extension view, (**C**) Lateral view, (**D**) Mouth opening view. Columns illustrate individual Grad-CAM visualizations for five different patients within the same view category, demonstrating the model's focus areas across various cases. Note: The sequence of images in each row does not imply any order or specific significance, as each column represents separate patient cases.

Gradient-weighted class activation mapping (Grad-CAM) proposes a technique that creates a “visual description” of the decision in many types of convolutional neural network (CNN)-based models, making it more transparent^15^. This flows into the final convolution layer to generate an approximate localization map that highlights important areas of the image to predict the concept using the slope of all target concepts. Grad-CAM can be applied to various CNN model families. When a patient with DDL is predicted to have DDL, Fig. 3 shows Grad-CAM according to the picture views in patients with high probability of DDL.

## Discussion

We developed a deep learning model capable of predicting DDL, indicative of challenging airway cases. We initially collected 18,163 photographs from 3,053 patients. After undersampling, the dataset for deep learning comprised 6,616 pictures from 1,288 patients. This model was trained and cross-validated using these 6,616 images, focusing exclusively on four types of pictures. The model's performance in the ROC AUC was 0.81–0.88. These results indicate that our deep learning model has the potential to effectively predict DDL, aiding healthcare practitioners in anticipating and preparing for challenging airway scenarios.

Several AI models predict difficult airway cases. Conventional machine learning models using multiple parameters have shown good performance but are limited by validation issues and the complexity of measuring numerous parameters^11,12,14^. Recently, deep learning models like Hayasaka et al.’s have classified difficult airways using patient pictures with an accuracy of 80.5%, sensitivity of 81.8%, specificity of 83.3%, and an ROC AUC of 0.864^10^. Their model used tightly controlled imaging angles, which are hard to obtain in clinical practice. We propose a practical model for emergent situations with more flexible imaging setups. We propose a practical model for emergent situations with more flexible imaging setups. Additionally, their study uses the VGG-16 model, which has around 138 M parameters, making it slow for training and prediction^25^. The VGG-16's performance is also typically 3%–5% lower than that of modern CNN models^20^.

Connor et al. classified difficult intubations through computer facial analysis^26^. Traditional approaches for predicting difficult airway, largely based on physical assessments and measurements, have been pivotal yet exhibit variability in predictive accuracy due to subjective assessment criteria. Our study advances the field with a larger patient cohort and modern deep learning techniques, offering greater robustness and generalizability. Unlike earlier AI models using facial recognition or eigenfaces, our state-of-the-art deep learning methods significantly outperform these older techniques. This approach allows for accurate analysis of complex image data and addresses the realistic variability of clinical environments, ensuring broad applicability across diverse settings.

Tavolara et al. developed a method to identify difficult intubation cases from front-face images using CNN-based feature extractors and attention-based multiple instance learning^27^. However, two-step learning processes are generally less optimized than end-to-end deep learning frameworks^28^. In the context of DDL prediction, end-to-end deep learning frameworks have shown top performance in various image processing domains. Traditional handcrafted feature-based classification techniques, such as eigenface methods, are significantly outperformed by modern deep learning approaches, as evidenced by several benchmark studies^29–31^.

The performance of our model (ROC-AUC [0.82–0.86] and sensitivity [0.63–0.9]) was similar to the results of Hayasaka et al.^15^, who developed an artificial intelligence model for classifying difficult laryngoscopy using facial images (ROC-AUC [0.793–0.897] and sensitivity [0.727–0.9]). However, our approach has distinct advantages. Specifically, our method uses fewer photographs and general-purpose smartphone cameras, making it easier to implement in diverse clinical settings without the need for specialized equipment. This ease of use and adaptability in real-world clinical environments is particularly valuable for anesthesiologists who need quick and reliable predictions to manage patient airways effectively.

Our study evaluated three models based on different added data types. Beyond basic picture data, we included models using picture view information and practitioner experience. For predicting DDL, practitioner experience seemed less important as the ROC AUC was lower than that of models using picture view information. This suggests practitioner experience may be a confounding factor. Given that pictures clearly differ according to the kind of picture view, the information for that view may be obtained by the deep learning model itself. Prior to the study, training was conducted to standardize the procedure for tracheal intubation and the evaluation of the Cormack-Lehane grade. However, several factors could contribute to variability in the model's performance:

- Variability in Skill Level and Judgment: Diverse practitioner experience introduces variability in evaluating laryngoscopic views, which may not benefit the model's learning process.
- Subjectivity in Evaluating Airway Difficulty: Practitioners' subjective evaluations, based on experience and intuition, may not align with objective image criteria, potentially confounding model predictions."

In Grad-CAM images predicting DDL, heat maps frequently highlighted the neck or chin areas in frontal, frontal-extension, and lateral views. This aligns with practitioners' focus on neck and chin size, ratio, and angle for airway evaluation. However, in mouth view images, heat maps appeared on the lips or tongue, differing from practitioners' focus on the Mallampati grade, suggesting variation in important features between clinical and AI evaluations.

We categorized patients into DDL and NDDL using the widely accepted Cormack-Lehane grading system. While all patients were successfully intubated, some required alternative methods after an initial failed attempt to ensure patient safety. Although it is also important to assess various tools, such as the Mallampati score and instances of failed intubation via direct laryngoscopy, our study specifically focused on the Cormack-Lehane grading system observed during laryngoscopies conducted with a MAC blade. This focused approach ensured a consistent and standardized assessment methodology, prioritizing patient safety throughout the process.

This study highlights the complexities of predicting airway difficulty using the Cormack-Lehane grading system. While grade 3 or 4 views serve as approximate markers for potential challenges, intubation difficulties can also occur in patients with grade 1 or 2 views. These challenges may include multiple attempts, prolonged duration, or failure to intubate via laryngoscopy. This underscores the need for a comprehensive approach to airway assessment beyond laryngeal view grades.

Furthermore, the higher DDL rate in our study, exceeding the typical < 10% reported in the literature^32–34^, may be attributed to the older median patient age (58 years), which is associated with increased difficult laryngoscopy^35^. However, potential over-reporting of grade 3 and 4 views or misclassification of non-difficult patients could also contribute to this discrepancy. This limitation suggests the need for future studies to further standardize and validate the grading process for accurate classification.

In this study, we analyzed the distribution of difficult laryngoscopy (DL) cases across anesthesiologists' experience levels before and after under-sampling. Initially, DL cases were 20.1% for 1–2 years, 25.5% for 2–3 years, 25.3% for 3–4 years, 25% for 4–5 years, and 16% for over 5 years of experience. Post under-sampling, the proportions changed to 35.8% for 1–2 years, 45.8% for 2–3 years, 47.3% for 3–4 years, 27.8% for 4–5 years, and 34.6% for over 5 years. This increased representation of difficult cases is crucial for model training but raises concerns about overestimating DL occurrence in certain experience categories. Future research should involve more anesthesiologists to better understand experience-related variability and verify model robustness in clinical settings.

The primary strength of this study lies in its innovative application of a deep learning model to predict Difficult Direct Laryngoscopy (DDL) using a minimal set of four types of photographs taken at the bedside. This approach not only demonstrates the model's robust performance but also highlights its potential for easy implementation in clinical settings, making it a significant contribution to the field.

However, the study does have several limitations that warrant consideration:

- Limited Number of Picture Views: Our model was trained with only four picture views. Tracheal intubation difficulty is assessed through comprehensive observations, so incorporating additional views could provide a more holistic assessment. Future improvements could involve multimodal training with various data types, including voice, 3D data, and video.
- Application to Videolaryngoscopy: With video laryngoscopy becoming standard and reducing difficult laryngoscopies, it's crucial to evaluate our model's performance in these settings. Future studies should explore its effectiveness where DL occurrences are lower.
- Leveraging DDL Predictions for Comprehensive Airway Management: While our model demonstrates promising performance in predicting DDL, it is important to understand that predicting difficult airways involves various factors beyond laryngoscopy alone. DDL prediction does not necessarily equate to predicting other forms of difficult airway situations, such as difficult mask ventilation or difficult intubation. However, the knowledge and learned features from our deep learning model for predicting DDL can be leveraged to develop prediction models for other aspects of difficult airway management. By fine-tuning or adapting the pre-trained model, the learned representations can be utilized to improve the performance and generalization of new models for predicting other airway challenges, such as difficult mask ventilation or difficult intubation.
- Practitioner Experience and Comack Lehane grading Variability: This study acknowledges critical considerations regarding anesthesiologists' experience and laryngoscopy skill development. Setting the lowest experience category at 1–2 years may not fully capture the rapid skill acquisition during the initial training period. Additionally, the volume of intubations performed over a year or more can significantly enhance an anesthesiologist's experience, potentially affecting study outcomes. Our analysis did not account for this incremental experience gain. Despite the expectation that performance improves with experience, the incidence of DDL did not significantly vary with anesthesiologist experience in our study. This suggests our multi-task learning model may not effectively capture the impact of practitioner experience on DDL prediction. Future research should explore more nuanced models or additional factors to better account for experience variability and its effect on airway management outcomes.
- Single-center design and population diversity: Results from our single-center study may not apply universally, especially to regions with diverse ethnicities and physical characteristics. For instance, our study population, averaging 163 cm in height and 55.1% male, differs significantly from populations like Scandinavia, where the average male height exceeds 180 cm^36^. This limitation underscores the need for future research to validate the model's applicability across diverse populations.
- The Impact of Low Incidence Rates on Model Accuracy in Clinical Settings: In practical clinical settings, the occurrence of Difficult Direct Laryngoscopy (DDL) is significantly less common than what might be simulated or expected in a controlled study environment. Typically, DDL may occur in as few as 5% of cases, depending on various factors such as the patient population and the specific clinical setting. This infrequent occurrence significantly impacts the performance of predictive models used in research. The lower incidence rate of DDL in real-world settings can lead to a disproportionately higher number of false positives compared to true positives. This skew can degrade the specificity and positive predictive value (PPV) of a model. Moreover, despite Cormack and Lehane system's widespread use, the classification has significant limitations. It was not initially designed as a predictive tool for intubation difficulty, which often leads to inconsistencies in its application^37^. Variations in interpretation among anesthesiologists can result in differing assessments of the same airway view.
- Limited neuromuscular monitoring: One limitation of our study is the lack of uniform verification of muscle relaxation using neuromuscular monitoring techniques. This was due to the variable availability of neuromuscular monitoring equipment across operating rooms and specific patient conditions, such as upper limb surgeries or the presence of extensive monitoring equipment on the upper limbs. This variability might have influenced the consistency of muscle relaxation achieved before intubation.

Addressing these limitations highlights the need for ongoing research to expand the model's applicability, improve accuracy across diverse clinical settings, and refine its utility in predicting DDL and other aspects of difficult airway management. Future studies should build on this research to achieve these goals.

In conclusion, our model shows good performance despite using simple and limited data. It demonstrates the potential for practical use as a predictive model in clinical practice and can serve as a pre-trained model for future learning. Performance and coverage improvement, along with external validation, are required for its clinical application, and future studies should extend to predicting difficult airway intubation.

## Data Availability

The datasets generated and/or analyzed during the current study contain sensitive personal information, such as photographs of patients' faces, and therefore are not publicly available. The data can be accessed from the corresponding author upon reasonable request, following anonymization and approval by the data review committee.
